# High Pollination Deficit and Strong Dependence on Honeybees in Pollination of Korla Fragrant Pear, *Pyrus sinkiangensis*

**DOI:** 10.3390/plants11131734

**Published:** 2022-06-29

**Authors:** Qian Li, Mengxiao Sun, Yangtian Liu, Bing Liu, Felix J. J. A. Bianchi, Wopke van der Werf, Yanhui Lu

**Affiliations:** 1 State Key Laboratory for Biology of Plant Diseases and Insect Pests, Institute of Plant Protection, Chinese Academy of Agricultural Sciences, Beijing 100193, China; liqian362771034@163.com (Q.L.); smx19921117@126.com (M.S.); yangtianlwmt@163.com (Y.L.); liubing1945@126.com (B.L.); 2 Centre for Crop Systems Analysis, Wageningen University & Research, 6700 AK Wageningen, The Netherlands; 3 Farming Systems Ecology, Wageningen University & Research, 6700 AK Wageningen, The Netherlands; felix.bianchi@wur.nl

**Keywords:** hand pollination, *Apis mellifera*, flower visitation, fruit set, pollinator, wild bee

## Abstract

Pollination deficits can compromise fruit yield and quality and have been reported in several fruit crops. It is unknown whether there is a pollination deficit in the production of Korla fragrant pear, *Pyrus sinkiangensis*, in China, and if so, whether this deficit can be mitigated by the use of managed honeybees (*Apis mellifera*). We assessed insect communities, flower visitation, pollination deficit and honeybee contribution to pear pollination in Korla fragrant pear orchards in Xinjiang, China. Insect communities were monitored using colored pan traps, and pollination deficit was assessed by comparing fruit set with open pollination to that with hand pollination in orchards without beehives from 2018 to 2021. The contribution of honeybees to pollination was assessed by comparing flower visitation, fruit set and fruit quality in pear orchards with and without beehives in 2020 and 2021. In orchards without beehives, wild bees (72%) were the dominant pollinator group in pan traps, followed by honeybees (15%), moths, hoverflies, butterflies and wasps (Vespidae). Fruit set in these orchards was much lower with open pollination (8 ± 2%) than with hand pollination (74 ± 4%). When comparing pollination in orchards with and without beehives in 2020 and 2021, we found that honeybees were responsible for most of the flower visits in orchards with (96%) and without beehives (66%). Wild bees were responsible for 1% and 6% of flower visits in orchards with and without beehives, respectively. Fruit set was significantly higher in orchards with beehives (38 ± 9%) than in orchards without beehives (12 ± 3%), while fruit set and sugar content were positively associated with pollinator visitation rate. The findings reveal a large pollination deficit in Korla fragrant pear orchards, and show that this deficit can be mitigated using managed honeybees.

## 1. Introduction

Insect pollinators are essential for the pollination of many vegetable and fruit crops [1]. With the increasing demand for insect-pollinated crop products such as nuts, vegetables and fruits [2], and the reported declines in pollinators [3,4,5,6,7], there may be a risk of pollination deficits compromising crop yield and quality. However, such deficits are species- and region-specific [8,9,10]. For instance, Holland et al. [11] reported pollination deficits in sunflowers and oilseed rape but not in pears or pumpkins in Europe. In the USA, a pollination deficit was found in blueberry in Michigan, Oregon and British Columbia but not in Florida [10]. Pollinators and pollination therefore need to be studied for specific crops or varieties in their agro-ecological context.

The contribution of wild pollinators and honeybees to crop pollination and the associated yield and quality benefits are well documented, for instance, in apple [12,13], sweet cherry [14] and almond [15]. However, the relative effectiveness of honeybees and wild pollinator groups may be crop species-specific [4,12]. Species that require buzz pollination (e.g., tomato) cannot be pollinated by honeybees as they cannot produce the vibrations that are required to remove pollen [16,17,18]. In contrast, managed honeybees are the most important pollinators of several Rosaceous crops, such as pear [19] and avocado [20]. Furthermore, diverse pollinator communities may benefit crop pollination due to functional complementarity between pollinator species, and due to species interactions [21]. For example, wild pollinators enhanced the movement rate of honeybees and enhanced pollination effectiveness in almond [22], sunflower [23] and sweet cherry [24]. The implications of pollinator communities composed of managed honeybees and wild pollinators for crop pollination are not fully understood.

Like most Rosaceae, pear (*Pyrus communis*) is a self-incompatible entomophilous crop species that strongly depends on insect pollination from a compatible cultivar to set fruit [25]. However, pear flowers are not attractive for many pollinators because of the low sugar content of the nectar (often <10%) [26,27]. For instance, even though bumblebees and solitary bees are efficient pollinators, they often avoid pear trees [27,28]. Pear pollination therefore often relies on the use of managed honeybees, and in the absence of honeybees, insufficient pollination is a common cause of poor pear yields [26,27].

Korla fragrant pear (*Pyrus sinkiangensis* Yü) is a premium species of pear that is cultivated in Xinjiang, China, and is distinct from *Pyrus communis*. Based on its morphological characteristics, *P. sinkiangensis* is thought to be of complex hybrid origin involving P. communis and Chinese white pears (*P. bretschneideri*) [29]. Korla fragrant pear has high market value and is widely cultivated in the Korla oasis region in the (semi-)arid part of Xinjiang, China. This oasis region has an area of 7.3 × 10^5^ ha, and is characterized by intensively managed crops and few semi-natural habitats. Although largely undocumented, this region may be a difficult environment for pollinators because of the low wild floral resource availability during large parts of the year and the intensive use of insecticides. Moreover, because the region is surrounded by desert, it may experience a high level of isolation with limited exchange with pollinator communities outside of the region.

To ensure sufficient pollination, Korla fragrant pear growers often rely on artificial pollination by spraying a suspension of pollen in water, or they use managed honeybees. However, we lack information about the wild pollinator communities in Korla fragrant pear orchards. It is also unknown whether there is a pollination deficit in Korla fragrant pear, and how effective managed honeybees are in supporting the pollination of Korla fragrant pear. Therefore, we assessed the composition of pollinator communities in Korla fragrant pear orchards, and assessed whether there is a pollination deficit of Korla fragrant pear. We quantified the role of wild insects and managed honeybees as pear pollinators by assessing the number of flower visits, fruit set and fruit quality (seed set, fruit weight and sugar content) in Korla fragrant pear orchards with and without beehives.

## 2. Results

### 2.1. Monitoring of Insect Communities

**Figure 1:**
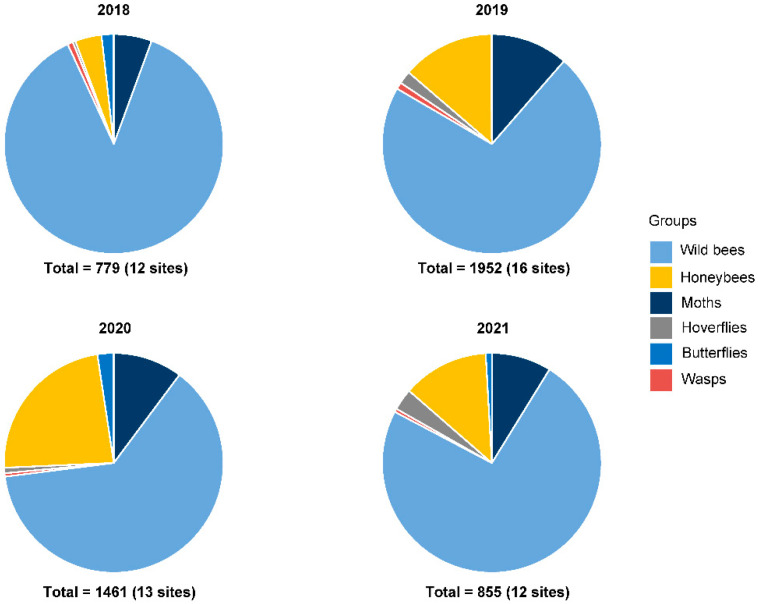
Number of individuals of six major groups of pollinating insects caught in pan traps in Korla fragrant pear orchards from 2018 to 2021.

We collected a total of 5047 insects in pan traps in 53 Korla fragrant pear orchards in 7–15-day sampling periods in four years. Wild bees were the most abundant insect group with 3638 individuals (72% of all specimens), followed by honeybees (745 individuals, 15% of all specimens) and moths (490 individuals, 10% of all specimens; Figure 1). Hoverflies, butterflies and wasps made up 4% of the sample with 79, 58 and 37 individuals, respectively (Figure 1).

### 2.2. Fruit Set

**Figure 2:**
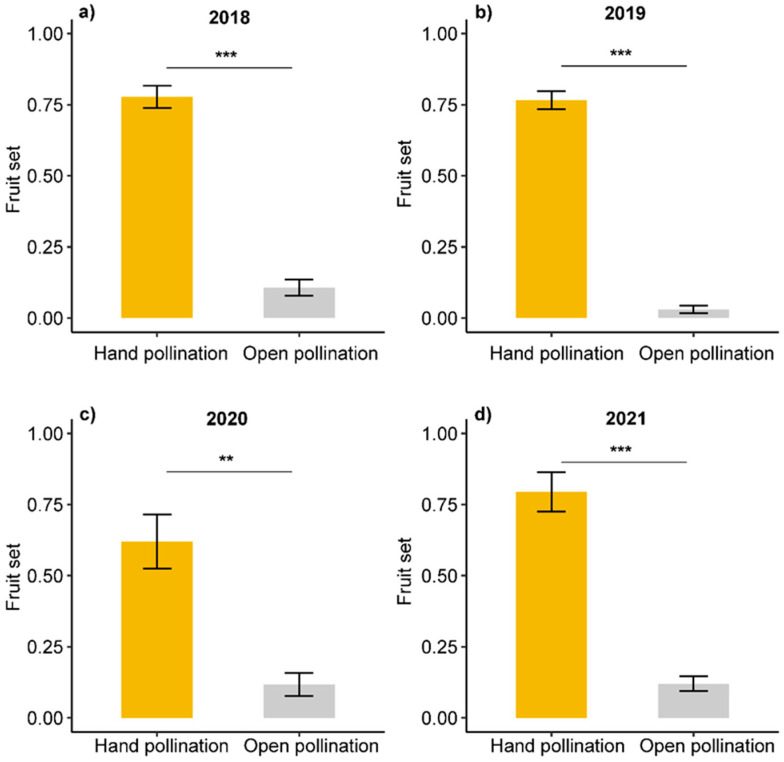
Fruit set in hand pollinated (yellow bars) and open pollinated Korla fragrant pear flowers (grey bars) in 2018 (**a**), 2019 (**b**), 2020 (**c**) and 2021 (**d**), respectively. Asterisks indicate significant differences (*** *p* < 0.001; ** *p* < 0.01) based on the results of a generalized linear model with quasibinomial error distribution (Table S1).

Fruit set was significantly lower in open pollinated pear trees (8 ± 2%) than in hand pollinated pear trees (74 ± 4%; *p* < 0.001), and this was consistent during the four study years as indicated by a non-significant year effect and year–treatment interaction (Figure 2, Table S1).

### 2.3. Flower Visitation, Fruit Set and Fruit Quality in Orchards with and without Beehives

**Figure 3:**
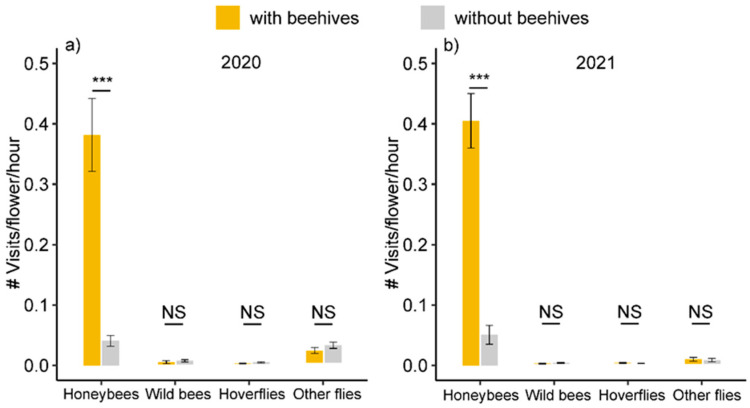
Flower visitation rates of honeybees, wild bees, hoverflies and other flies in Korla fragrant pear orchards with beehives (yellow bars) and without beehives (grey bars) in 2020 (**a**) and 2021 (**b**). Asterisks (***) indicate significant differences (*p* < 0.001) and NS indicates non-significant differences (*p* > 0.05) based on a linear model (Table S4).

We recorded in total 2207 flower visits in 41 orchards in 2020 and 2021, with 1981 honeybee visits, 36 wild bee visits, 13 hoverfly visits and 177 visits by other flies. No visits by other insects were recorded. Honeybees accounted for 96% and 66% of all visits in orchards with and without beehives. Honeybee visitation was significantly higher in orchards with beehives than in orchards without beehives in both years (0.38 ± 0.06 vs. 0.04 ± 0.01 visits/flower/hour in 2020 and 0.41 ± 0.05 vs. 0.05 ± 0.02 in 2021, *p* < 0.001), but there was no significant difference in the visitation rate of honeybees between 2020 and 2021. There was no significant effect of the presence of beehives (treatment) on the visitation rate of wild bees, hoverflies or other flies, but the visitation rate of hoverflies and other flies was significantly higher in 2020 than in 2021 (*p* = 0.029 for hoverflies, *p* < 0.001 for other flies; Figure 3, Tables S2 and S3).

**Figure 4:**
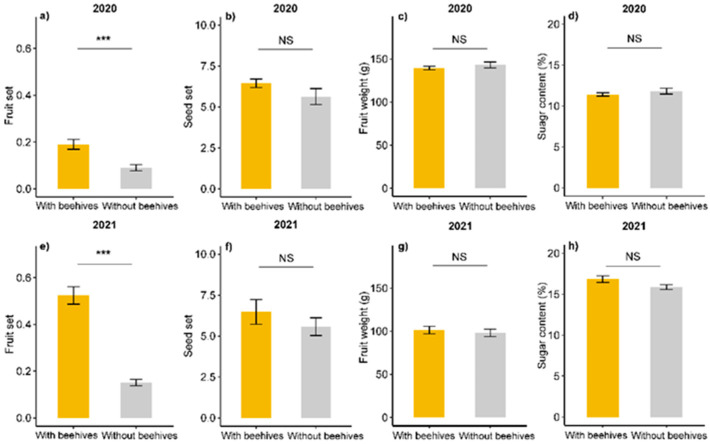
Fruit set (**a**,**e**), seed set (**b**,**f**), fruit weight (**c**,**g**) and sugar content (**d**,**h**) in Korla fragrant pear orchards with beehives (yellow bars) and without beehives (grey bars) in 2020 (**a**–**d**) and 2021 (**e**–**h**). Asterisks (***) indicate significant differences (*p* < 0.001) and NS indicates non-significant differences (*p* > 0.05) based on the results of a generalized linear model with quasibinomial error distribution for fruit set and linear models for seed set, sugar content and fruit weight (Table S7).

Fruit set was significantly higher in pear orchards with beehives than in orchards without beehives (*p* < 0.001), and fruit set was significantly higher in 2021 than in 2020 (*p* = 0.006; Figure 4; Tables S5 and S6). There was a significant interaction between the effect of beehives and year, indicating that fruit set in pear orchards with beehives was higher in 2021 than in 2020 (*p* = 0.001; Tables S5 and S6). The presence of beehives did not significantly influence seed set, fruit weight and sugar content. Sugar content was significantly higher in 2021 than in 2020 (*p* < 0.001), while fruit weight was significantly lower in 2021 than in 2020 (all *p* < 0.001; Figure 4, Tables S5 and S6).

**Figure 5:**
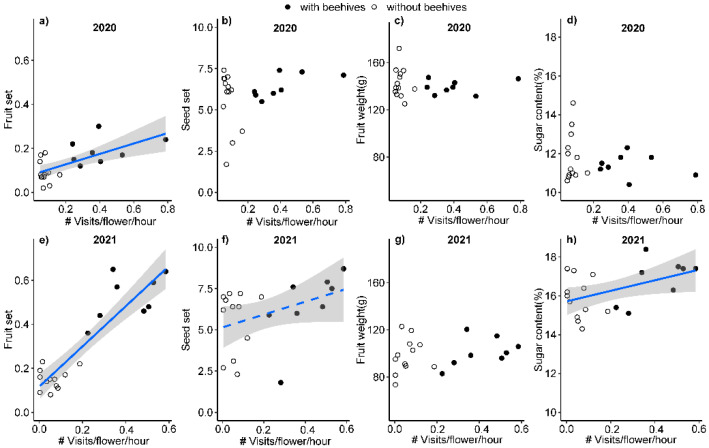
Relationship between total pollinator visitation rate of honeybees, wild bees, hoverflies and other flies, and fruit set (**a**,**e**), seed set (**b**,**f**), fruit weight (**c**,**g**) and sugar content (**d**,**h**) in Korla fragrant pear orchards in 2020 (**a**–**d**) and 2021 (**e**–**h**). Solid and dashed lines indicate significant *p* < 0.05) and marginally significant relationships (*p* < 0.1). Relationships are based on results from a generalized linear model with quasibinomial error distribution for fruit set, and from linear models for seed set, sugar content and fruit weight. Open symbols represent data from orchards without beehives, while closed symbols are data from orchards with beehives.

Fruit set was positively related with the total visitation rate of honeybees, wild bees, hoverflies and other flies in 2020 (*p* = 0.002) and 2021 (*p* < 0.001; Figure 5, Table S8). Seed set had a weak relationship with the total pollinator visitation rate in 2021 (*p* = 0.088) but not in 2020 (Figure 5, Table S8). Sugar content was positively related with the total pollinator visitation rate in 2021 (*p* = 0.036) but not in 2020 (Figure 5, Table S8). Fruit weight was not significantly related with total pollinator visitation in either year (Figure 5, Table S8).

Fruit set was positively related with the visitation rate of honeybees (*p* = 0.002 in 2020, *p* < 0.001 in 2021) but not with the visitation rate of wild bees, hoverflies or other flies in the two years (Table S8). Seed set had a weak positive relationship with the visitation rate of honeybees (*p* = 0.090) and wild bees (*p* = 0.071) in 2021, but seed set was not significantly related to the visitation rate of honeybees and wild bees in 2020, or with the visitation rate of hoverflies or other flies in 2020 or 2021. Sugar content was positively related with the visitation rate of honeybees in 2021 (*p* = 0.038) but not in 2020, and sugar content was not significantly related to the visitation rate of wild bees, hoverflies or other flies in either year. Fruit weight was not significantly related with the visitation rate of honeybees, wild bees, hoverflies and other flies in the two years (Table S8).

## 3. Discussion

In this study we assessed the adequacy of pollination in Korla fragrant pear and found fruit set to be low. This was shown by the relatively large difference in fruit set between open pollination (8%) and hand pollination (74%) in orchards without beehives, and the higher fruit set in orchards with beehives (38%) compared to orchards without beehives (8%). While wild bees made up more than 70% of the insects collected in pan traps, fewer than 2% of the recorded pear flower visits were made by wild bees. Instead, honeybees made the majority of flower visits in orchards with beehives (96%) and without beehives (66%). In the orchards without beehives, flies were the second most frequent group of flower-visiting insects (26%). Flower visitation by pollinators was positively associated with fruit set and also with pear sugar content in 2021. Thus, our study shows low pollination success in Korla fragrant pear orchards (as measured by fruit set) unless honeybee beehives were placed to supplement pollination by naturally occurring pollinators.

While we found a clear pollination deficit in Korla fragrant pear in Xinjiang, reports on pollination deficits in European pear (*Pyrus communis*) are mixed. Pollination deficits have been reported for the varieties “Conference” (21% in open pollination vs. 30.7% in hand pollination) and “Doyenne du Comice” (7.2% in open pollination vs. 16.8% in hand pollination) in Belgium [28], while Holland et al. [11] found no consistent pollination deficit in “Conference” pear in the Netherlands. The absence or relatively low pollination deficit in “Conference” pear may be due to its capacity to produce fruit by spontaneous parthenocarpy, which buffers against a low pollinator visitation rate [30]. In Korla fragrant pear, the high pollination deficit underlines the lack of effective pollinators and explains why farmers are looking for ways to enhance pollination and regularly resort to artificial pollination.

When comparing pear pollination in orchards with and without beehives, we found honeybee was the dominant pear flower visitor in orchards with (96%) and without beehives (66%), and few wild bees were observed to visit pear flowers. Similar findings have been reported in pear orchards in Argentina [31], apple orchards in Germany [32] and Macadamia in South Africa [33] where honeybees were the major visiting flower pollinator and wild pollinators were virtually absent. In addition, our results confirmed that orchards with beehives had 6.0-fold higher pear flower visits and 3.2-fold higher fruit set than orchards without beehives, and that these effects were consistent across two years. Flower visitation was also positively associated with pear sugar content and seed set, but this was only observed in one out of two years. Positive effects of honeybees on fruit set and fruit quality have also been found in kiwi [34] and blueberry [35]. Establishing beehives is a relatively easy strategy to sufficiently enhance pear flower visitation, fruit quality and yield. Flower visitation does not necessarily imply pollination because there is a great variation in effectiveness among pollinators [36]. Nevertheless, our analysis of the relationships between flower visits by different insect groups and fruit set (Table S8) provides clear evidence that honeybees are mainly responsible for pollination in Korla fragrant pear. Enhancing wild pollinator communities will be more challenging, particularly in intensively managed pear production landscapes, and may require an integral insect conservation strategy [37]. Even if such conservation efforts are successful, a higher abundance and diversity of wild pollinators is no guarantee for better pollination of Korla fragrant pear because many wild pollinator species are reluctant to visit pear flowers.

This is the first study showing that there is a large pollination deficit in Korla fragrant pear and that introducing beehives can sufficiently alleviate the pollination deficit and contribute to improved fruit quality in Korla fragrant pear. Pear growers are aware of the pollination deficit because they invest in hiring beehives and in artificial pollination. Hand pollination is conducted with long sticks with a mesh with pollen at the end. The cost is around CNY 80 per mu (1/15th ha) per round of hand pollination (including helpers and pollen). For bee pollination, the local farmers usually place one to two beehives per mu, and each beehive costs around CNY 100, so the cost of bee pollination is around CNY 100–200 per mu. An advantage of bee pollination is that it is effective over the whole flowering period, if the weather is suitable [38]. On the other hand, hand pollination can only pollinate those flowers that are open at the time of a single round of hand pollination. The fact that farmers use both methods of supplementary pollination (beehives and hand pollination) in practice indicates that the two methods are approximately equally attractive to them. Our observed increase in pear fruit set from 12 to 38% by introducing beehives should be sufficient to attain maximum yield since growers will typically thin the fruit to 2–3 fruits per flower cluster (usually 7~8 flowers), while a fruit set of 12% is not sufficient to attain maximum pear yield. However, the reliance on honeybees as a single pollinator species can be risky because honeybee is susceptible to parasites (e.g., varroa mite), diseases and insecticides [39]. Honeybee colony collapses have occurred in the past and may well happen again. Moreover, honeybees may compete with wild pollinators for floral resources [40,41]. Therefore, the call for restoring biodiversity-friendly landscapes [42] may certainly also be relevant for Korla fragrant pear production landscapes, which strongly depend on pollinators to support food production and livelihoods.

## 4. Materials and Methods

### 4.1. Study Sites and Experimental Design

The study was conducted in Korla, Xinjiang, north-west China (E 85.48°, N 41.45°), a prime production region of Korla fragrant pear. The pear trees in the selected orchards were 15–20 years old and in full production. The orchards consisted of Korla fragrant pear trees mixed with Dangshan pear (*P. bretschneideri*) trees, which is used as pollinizer but whose fruit have low market value. The orchards were embedded in landscapes that were dominated by pear orchards and cotton, but also comprised maize, peach, apricot and jujube, and about 7% semi-natural habitat (range 0.5–14% within a 2 km radius around fields). Pears are frequently treated with insecticides throughout the growing season (e.g., usually once per 2–4 weeks), except from one week before flowering until the end of flowering to avoid direct impacts on pollinators. There are no wild honeybees in Korla.

Two series of measurements were set up (Table 1). Measurement series 1 ran over four years (2018–2021) and involved measuring the insect community in orchards without beehives using pan traps and quantifying pollination deficit by comparing the fruit set between open pollinated flowers to that of hand pollinated flowers as a reference. In measurement series 2, conducted in 2020 and 2021, we compared the visitation rate of pollinators, fruit set and fruit quality between orchards with and without beehives. Orchards without beehives were used for both types of measurements in 2020 and 2021.

### 4.2. Monitoring of Insect Communities

We used pan trapping to assess insect communities in 53 Korla fragrant pear orchards without beehives during flowering for four years (measurement series 1; Table 1). Each year, new orchards were selected. Insect communities were assessed using colored pan traps [43]. Pan trap stations consisted of three cups (12.1 cm diameter, 13 cm height) that were painted ultraviolet (UV) yellow (SANO, type No. 1005), UV blue (SANO, type No. 1004) or UV white (SANO, type No. 1010) on both the inside and the outside, and that were fixed on three different branches on the same pear tree at approximately 1.5 m height. Four trap stations were arranged on the corners of a 50 by 50 m square in the middle of pear orchards, and stations were located at least 20 m from the edge of the orchard. Sampling was conducted during the flowering period of pear in the first half of April for a period of 7–15 days with traps being emptied at approximately 3-day intervals (13–20 April 2018, 3–18 April 2019, 5–15 April 2020 and 2–14 April 2021, respectively). The total insect catch in each orchard was sorted into six main groups: honeybees (*Apis mellifera*), wild bees (e.g., Halictidae, Sphecidae, and Melittnae), hoverflies (mostly *Syrphus corollae* and *Episyrphus balteatus*), butterflies (mostly *Pieris rapae*), wasps (e.g., *Vespula germanica*) and moths (mostly *Grapholitha molesta*) as these were considered to be potential pollinators [30]. Other insects in traps, such as other Diptera, Hymenoptera (e.g., Cephidae), Coleoptera (e.g., Coccinellidae and Meloidae), Neuroptera (e.g., Chrysopidae), Hemiptera (e.g., Miridae) were not considered.

### 4.3. Pollination Deficit Assessment

The pollination deficit was assessed in a subset of 30 out of the 53 Korla fragrant pear orchards mentioned above (11, 5, 4 and 10 orchards in 2018, 2019, 2020 and 2021, respectively) by comparing the fruit set between open pollinated flowers and hand pollinated flowers. At each site, two similar trees were randomly selected at the center of the orchard. One tree was used to measure fruit set with open pollination and the other was used to measure fruit set with hand pollination. Ten branches were randomly selected on each tree, and from each branch one flower cluster was labelled. Each cluster was standardized to three newly open flowers by carefully removing excess flowers by hand. The open pollination treatment did not receive any treatment and therefore depended on naturally occurring pollinators. The flowers of the hand pollination treatment received commercially available pollen of Dangshan pear, which were put on flower stigmas using a cotton pad. The standardization of flower clusters and hand pollination was conducted on 9 April 2018, 8 April 2019, 11 April 2020 and 9 April 2021, and the number of fruitlets on hand and open pollinated trees was recorded on 28 April 2018, 25 April 2019, 26 April 2020, 25 April 2021, respectively.

### 4.4. Flower Visitation, Fruit Set and Fruit Quality in Orchards with and without Beehives

The contribution of honeybees to pear pollination was assessed in 2020 and 2021. Eight orchards with beehives (treatment) were selected in 2020 and 2021, while 13 orchards without beehives (control) were selected in 2020 and 12 control orchards were selected in 2021. In both years, the orchards with beehives were newly selected, while the orchards without beehives were the same as those used in measurement series 1. In these 41 orchards we measured flower visitation, fruit set and fruit quality (seed set, fruit weight and sugar content). Orchards with and without beehives were located at least 2 km apart.

We assessed flower visitation by visual observation during full bloom. Observations were conducted at four time periods per day (10:00–11:30, 12:00–13:30, 14:00–15:30 and 16:00–17:30) under dry weather conditions with temperatures ranging between 10 °C and 22 °C and wind speeds below 30 km/h. Four observers were involved. For each observation round, four trees were randomly selected in the center of the orchard. On each selected tree, one branch with an approximate diameter of 1 cm was chosen at around 1.5 m height. A group of 100 open flowers on the branch was marked as the observation area. During a 10 min-observation period, we recorded the number of insects visiting, and for each insect we recorded the number of flowers visited in the observation area. A visit was defined as the insect making contact with the stigma of a flower. The flower visitors were sorted into four groups: honeybees (*Apis mellifera*), wild bees, hoverflies and other flies. We did not find other insects visiting flowers except for from these four groups. In total, four observation rounds were conducted per orchard, one at each of the four designated times, and we selected four new trees for each observation round. With four observers and four times of observation, the total observation time per orchard was 160 min, and all visits of insects in each flower visitor group per orchard were pooled. For each pollinator group, the pollinator visitation rate per orchard was expressed as number of visits per flower per hour. Trees selected for pollinator observations in orchards without beehives were always at least 10 m apart from trees with pan traps to avoid pollinator depletion near the traps.

Fruit set was defined as the proportion of fruitlets per flower cluster before thinning (i.e., initial fruit set), which started at the end of April [19]. During pear flowering, ten trees were selected per orchard in an “X” pattern. For each tree, three 1 to 2-year-old branches were selected in each of four cardinal directions, and one flower cluster per branch was marked with a piece of red string, for a total of 12 flower clusters per tree. The number of flowers per marked cluster was standardized to three. The number of fruitlets per marked cluster was recorded one week after the end of flowering (before thinning). The fruit set rate for each orchard was calculated as the average proportion fruitlets developing from the initial number of marked flowers per tree.

We used three indicators of fruit quality: seed set (which is associated with pear shape, calcium concentration and flesh firmness), fruit weight and sugar content. These were assessed in early September, shortly before commercial harvest. Six pears were randomly selected from five trees separately in an “X” pattern for a total of 30 pears per orchard. Pears were weighed on an electronic balance, and the number of mature, black seeds was counted. The sugar concentration of the pear juice was measured with a mini digital display sugar meter (Product name: Pocket Refractometer; Type: PAL-1; Manufacturer: ATAGO).

### 4.5. Statistical Analysis

Three types of analyses were conducted. Firstly, the insects caught in pan traps were classified into six groups (honeybees, wild bees, moths, butterflies, hoverflies and wasps) to determine the insect community composition in each year. Secondly, the influence of hand pollination and open pollination on fruit set (response variable) was analyzed with a generalized linear model (glm) with quasibinomial error distribution to account for overdispersion. Explanatory variables were treatment (hand versus open pollination), year and the interaction between treatment and year. Furthermore, glms were fitted for separate years with treatment (hand versus open pollination) as the only predictor variable. Thirdly, two analyses were conducted to determine the effect of beehives on pollinator visitation rate and pollination service (i.e., fruit set, fruit weight, seed set and sugar content). On the one hand, we analyzed the influence of presence or absence of beehives (treatment) on pollinator visitation rate and metrics for pollination service. Response variables included flower visitation rates of honeybees, wild bees, hoverflies and other flies (square root transformed data for all groups and normal error distribution), fruit set (quasibinomial error distribution), seed set, fruit weight and sugar content (all using normal error distribution). Explanatory variables were presence or absence of beehives (treatment), year and the interaction between treatment and year. We used linear models for variables with a normal error distribution and a glm with quasibinomial error distribution for fruit set to account for overdispersion. For year-specific analyses we used the same models with treatment as the only explanatory variable. On the other hand, the relationship between pollinator visitation rate and metrics for pollination services was explored using linear models and generalized linear models. Response variables included fruit set (quasibinomial error distribution), seed set, fruit weight and sugar content (all normal distribution), and the explanatory variables were visitation rate of total pollinators, honeybees, wild bees, hoverflies and other flies, respectively. The total pollinator visitation rate was calculated as the sum of the visitation rates of honeybees, wild bees, hoverflies and other flies per orchard.

All calculations and analyses were performed in R [44]. The glms were fitted using the function glm() from the “MASS” package and linear models were fitted with lm() from the “stats” package. Model validation was conducted by visual inspection of the plotted residuals versus the predicted values and QQ plots [45]. Means and standard errors of the mean are reported throughout the text and figures.

## Figures and Tables

**Table 1:** Overview of treatments, observations and number of Korla fragrant pear orchards from 2018 to 2021.

Measurement Series	Year	Orchard Level Treatments	Measurements	No. of Orchards
1	2018	No beehives	Pan trapping Open and hand pollination	12 11
	2019	No beehives	Pan trapping Open and hand pollination	16 5
	2020	No beehives	Pan trapping Open and hand pollination	13 * 4
	2021	No beehives	Pan trapping Open and hand pollination	12 * 10
2	2020	With beehives	Flower visitation Fruit set and quality	8
	2020	No beehives	Flower visitation Fruit set and quality	13 *
	2021	With beehives	Flower visitation Fruit set and quality	8
	2021	No beehives	Flower visitation Fruit set and quality	12 *

* Shared orchards for measurement series 1 and 2.

## Data Availability

Not applicable.

## Supplementary Materials

The following supporting information can be downloaded at: https://www.mdpi.com/article/10.3390/plants11131734/s1, Figure S1: Year-specific analysis of the relationship between fruit set (response variable) and treatment (hand pollination versus open pollination); Table S2. Pollinator visitation rates of honeybees, wild bees, hoverflies and other flies (mean ± standard error) in pear orchards with (treatment) and without beehives (control) in 2020 and 2021; Table S3. Relationship between visitation rates of honeybees, wild bees, hoverflies and other flies (sqrt transformed; response variables) and treatment (presence or absence of beehives in the orchard) and year; Table S4. Year-specific analysis of the relationship between visitation rates of honeybees, wild bees, hoverflies and other flies (sqrt transformed; response variables) and treatment (presence or absence of beehives in the orchard); Table S5. Mean and standard error of pollination service in pear orchards with and without beehives (control) in 2020 and 2021; Table S6. GLM analysis of the effect of beehives on pollination services in pear orchards in 41 sites in 2020 and 2021 (in 2020: 8 sites with beehives and 13 sites without beehives; in 2021: 8 sites with beehives and 12 sites without beehives); Table S7. Year-specific analysis of GLM and LM analysis of the effect of managed honeybee hives on pollination service in pear orchard in 41 sites in 2020 and 2021 (in 2020: 8 sites with beehives and 13 sites without beehives; in 2021: 8 sites with beehives and 12 sites without beehives); Table S8. Generalized linear model (GLM) and linear model (LM) analysis of the relationship between pollination services and visitation rate of pollinators in pear orchards with and without beehives in 2020 and 2021. Figure S1. Korla fragrant pear tree with yellow, blue and white pan traps to sample the insect community around the tree.
