# Supplementary material for: High Pollination Deficit and Strong Dependence on Honeybees in Pollination of Korla Fragrant Pear, Pyrus sinkiangensis
Source: Plants (Basel). 2022 Jun 29;11(13):1734. doi: 10.3390/plants11131734 (PMC9268985; doi:10.3390/plants11131734)
Supplement: Supplementary file 1 [file plants-11-01734-s001.zip › plants-1752262-supplementary.pdf]

Supplementary Data

High pollination deficit and strong dependence on honeybees in pollination of Korla fragrant pear, *Pyrus sinkiangensis*

Qian Li[1,2], Mengxiao Sun[1,2], Yangtian Liu[1], Bing Liu[1], Felix J. J. A. Bianchi[3], Wopke van der Werf[2*] and Yanhui Lu[1*]

Table S1. Year-specific analysis of the Relationship between fruit set (response variable) and treatment (hand pollination versus open pollination). Each line in the table specifies the effect of hand pollination (on the logit scale). A GLM with binomial error distribution was used. Open pollination was considered as reference.

	Estimate	Std. Error	t value	*P* value
2018	3.38	0.38	8.91	**<0.001**
2019	4.74	0.50	9.55	**<0.001**
2020	2.49	0.61	4.11	***0.006***
2021	3.35	0.53	6.37	**<0.001**

Table S2. Pollinator visitation rates of honeybees, wild bees, hoverflies and other flies (mean ± standard error) in pear orchards with (treatment) and without beehives (control) in 2020 and 2021. Coefficients are reported * 1000.

	2020		2021	
	Treatment (*1000)	Control (*1000)	Treatment (*1000)	Control (*1000)
**Pollinator visitation rate** (***Number of Visits/ flower/ hour***)				
Honeybees	382±602	40.7±8.9	405±45	50.6±15.6
Wild bees	3.75±2.35	6.06±2.06	0.47±0.47	1.88±0.73
Hoverflies	0.94±0.61	2.60±1.15	0.94±0.94	0.00±0.00
Other flies	19.7±4.9	28.6±5.3	7.50±3.17	6.25±2.78

Table S3. Relationship between visitation rates of honeybees, wild bees, hoverflies and other flies (sqrt transformed; response variables) and treatment (presence or absence of beehives in the orchard) and year. A linear model was used, and pear orchards without beehives (control) in 2020 was taken as the reference.

	Estimate (*1000)	Std. Error (*1000)	z value	*P* value
**Pollinator visitation rate** (# ***Visits/ flower/ hour***)				
1) Honeybees				
Beehives	418.23	51.58	8.11	**<0.001**
Year	-8.61	46.00	-0.19	0.852
Beehives*Year	31.70	73.52	0.43	0.669
2) Other flies				
Beehives	-28.30	26.10	-1.08	0.285
Year	-100.56	23.25	-4.33	**<0.001**
Beehives*Year	35.30	37.20	0.95	0.349
3) Wild bees				
Beehives	-19.26	20.38	-0.95	0.351
Year	-27.23	18.16	-1.50	0.142
Beehives*Year	-0.71	29.05	-0.03	0.981
4) Hoverflies				
Beehives	-12.84	13.95	-0.92	0.363
Year	-28.14	12.42	-2.27	**0.029**
Beehives*Year	23.66	19.88	1.19	0.242

Table S4. Year-specific analysis of the relationship between visitation rates of honeybees, wild bees, hoverflies and other flies (sqrt transformed; response variables) and treatment (presence or absence of beehives in the orchard). A linear model was used, and pear orchard without beehives (control) was taken as the reference.

	Estimate	Std. Error	t value	*P* value
1) Honeybees				
2020	0.42	0.04	9.35	**<0.001**
2021	0.45	0.06	7.64	**<0.001**
2) Other flies				
2020	-0.03	0.03	-1.08	0.294
2021	0.01	0.03	0.27	0.794
3) Wild bees				
2020	-0.02	0.03	-0.77	0.453
2021	-0.02	0.01	-1.44	0.167
4) Hoverflies				
2020	-0.01	0.02	-0.73	0.474
2021	<0.01	<0.001	1.24	0.230

Table S5. Mean and standard error of pollination service in pear orchards with and without beehives (Control) in 2020 and 2021.

	2020		2021	
	Beehives	Control	Beehives	Control
Fruit set	0.19±0.02	0.09±0.01	0.52±0.04	0.15±0.01
Seed set	6.44±0.25	5.63±0.49	6.48±0.75	5.57±0.54
Sugar content	11.40±0.21	11.82±0.34	16.84±0.40	15.88±0.30
Fruit weight	139±2	143±3	101±4	98.1±4.3

Table S6. GLM analysis of the effect of beehives on pollination services in pear orchards in 41sites in 2020 and 2021 (in 2020: 8 sites with beehives and 13 sites without beehives; in 2021:8 sites with beehives and 12 sites without beehives). The response variables were initial fruit set (Quasibinomial error distribution), seed set (Normal error distribution), fruit weight (Normal error distribution) and sugar content (Normal error distribution). We used GLM was applied for fruit set and LM for seed set, sugar content and fruit weight. The explanatory variables with or without beehives and year. Pear orchards without beehives (control) in 2020 were taken as reference.

	Estimate	Std. Error	t value	*P* value
**1) Fruit set**				
Beehives	0.87	0.21	4.07	**<0.001**
Year	0.59	0.20	2.90	**0.006**
Beehives*Year	0.95	0.28	3.44	**0.001**
**2) Seed set**				
Beehives	0.81	0.78	1.04	0.307
Year	-0.06	0.69	-0.09	0.927
Beehives*Year	0.10	1.11	0.09	0.928
**3) Sugar content**				
Beehives	-0.42	0.48	-0.87	0.388
Year	4.06	0.42	9.58	**<0.001**
Beehives*Year	1.38	0.68	2.03	*0.050*
**4) Fruit weight**				
Beehives	-3.64	5.47	-0.67	0.510
Year	-44.98	4.87	-9.23	**<0.001**
Beehives*Year	6.83	7.79	0.88	0.386

Table S7. Year-specific analysis of GLM and LM analysis of the effect of managed honeybee hives on pollination service in pear orchard in 41 sites in 2020 and 2021 (in 2020: 8 sites with beehives and 13 sites without beehives; in 2021: 8 sites with beehives and 12 sites without beehives). The response variables were initial fruit set (Quasibinomial error distribution), seed set (Normal error distribution), fruit weight (Normal error distribution) and sugar content (Normal error distribution). The explanatory variables with or without beehives (treatment). GLM was applied for fruit set and LM was applied for seed set, sugar content and fruit weight. Pear orchards without beehives (control) were taken as reference.

	Estimate	Std. Error	t value	*P* value
1) Fruit set				
2020	0.87	0.21	4.13	**<0.001**
2021	1.82	0.18	10.32	***<0.001***
2) Seed set				
2020	0.81	0.66	1.23	0.234
2021	0.91	0.91	1.01	0.328
3) Suagr content				
2020	-0.42	0.47	-0.89	0.386
2021	0.96	0.49	1.96	*0.066*
4) Fruit weight				
2020	-3.64	4.68	-0.78	0.446
2021	3.19	6.29	0.51	0.618

Table S8. Generalized linear model (GLM) and linear model (LM) analysis of the relationship between pollination services and visitation rate of pollinators in pear orchards with and without beehives in 2020 and 2021. The response variables were fruit set (GLM; Quasibinomial error distribution), seed set (LM; Normal error distribution), sugar content (LM: Normal error distribution) and fruit weight (LM; Normal error distribution). The explanatory variables were visitation rate of total pollinators, honeybees, wild bees, hoverflies and other flies.

		Estimate	Std. Error	t value	Pr(>|t|)
**1) Fruit set (GLM)**					
2020	Total	1.81	0.51	3.56	**0.002**
	Honeybees	1.83	0.51	3.61	**0.002**
	Wild bees	-12.40	21.42	-0.58	0.570
	Hoverflies	-23.98	44.00	-0.52	0.607
	Other flies	-1.91	8.44	-0.23	0.823
2021	Total	4.37	0.53	8.17	**<0.001**
	Honeybees	4.39	0.53	8.23	**<0.001**
	Wild bees	-148.15	110.85	-1.34	0.198
	Hoverflies	204.34	120.28	1.70	0.107
	Other flies	8.51	24.98	0.36	0.727
**2) Seed set (LM)**					
2020	Total	2.11	1.65	1.28	0.217
	Honeybees	2.05	1.64	1.25	0.225
	Wild bees	-5.66	48.0	-0.12	0.907
	Hoverflies	13.63	97.32	0.14	0.890
	Other flies	1.99	19.44	0.10	0.919
2021	Total	3.89	2.15	1.81	*0.088*
	Honeybees	3.88	2.17	1.79	*0.090*
	Wild bees	-370.89	193.18	-1.92	*0.071*
	Hoverflies	388.77	262.87	1.48	0.156
	Other flies	30.91	50.48	0.61	0.548
**3) Sugar content (LM)**					
2020	Total	-1.09	1.18	-0.92	0.369
	Honeybees	-1.04	1.17	-0.89	0.387
	Wild bees	-16.00	33.44	-0.48	0.638
	Hoverflies	28.95	67.92	0.43	0.675
	Other flies	-2.13	13.62	-0.16	0.877
2021	Total	2.72	1.21	2.26	**0.036**
	Honeybees	2.72	1.21	2.24	**0.038**
	Wild bees	-184.02	115.79	-1.59	0.129
	Hoverflies	160.00	157.89	1.01	0.324
	Other flies	21.53	29.31	0.74	0.471
**4) Fruit weight (LM)**					
2020	Total	-6.63	11.91	-0.56	0.585
	Honeybees	-6.67	11.79	-0.57	0.578
	Wild bees	444.51	319.00	1.39	0.180

	Hoverflies	-144.09	678.64	-0.21	0.834
	Other flies	-44.49	135.30	-0.33	0.746
2021	Total	15.88	15.52	1.02	0.320
	Honeybees	15.04	15.64	0.96	0.349
	Wild bees	-984.02	1427.41	-0.69	0.499
	Hoverflies	896.84	1886.81	0.48	0.640
	Other flies	500.68	327.23	1.53	0.143

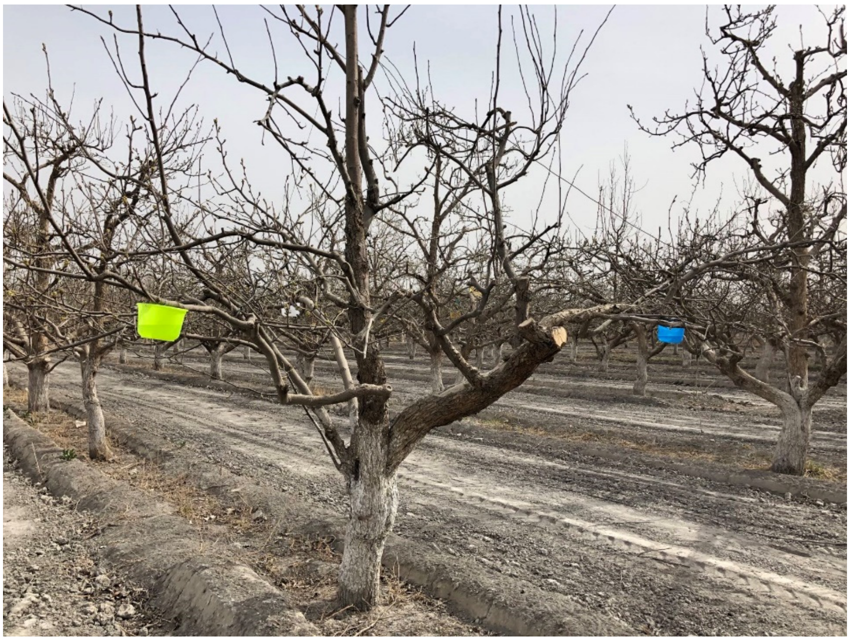

Figure S1. Korla fragrant pear tree with yellow, blue and white pan traps to sample the insect community around the tree. Picture taken shortly before flowering.
